# A comparison of baseline methodologies for 'Reducing Emissions from Deforestation and Degradation'

**DOI:** 10.1186/1750-0680-4-4

**Published:** 2009-07-13

**Authors:** Michael Huettner, Rik Leemans, Kasper Kok, Johannes Ebeling

**Affiliations:** 1 Max-Planck-Institute for Biogeochemistry, Hans-Knöll-Str 10, 07745 Jena, Germany; 2 University of Wageningen, PO Box 47, 6700 AA Wageningen, the Netherlands; 3 EcoSecurities, 40-41 Park End Street, Oxford, OX1 1JD, UK

## Abstract

**Background:**

A mechanism for emission reductions from deforestation and degradation (REDD) is very likely to be included in a future climate agreement. The choice of REDD baseline methodologies will crucially influence the environmental and economic effectiveness of the climate regime. We compare three different historical baseline methods and one innovative dynamic model baseline approach to appraise their applicability under a future REDD policy framework using a weighted multi-criteria analysis.

**Results:**

The results show that each baseline method has its specific strengths and weaknesses. Although the dynamic model allows for the best environmental and for comparatively good economic performance, its high demand for data and technical capacity limit the current applicability in many developing countries.

**Conclusion:**

The adoption of a multi-tier approach will allow countries to select the baseline method best suiting their specific capabilities and data availability while simultaneously ensuring scientific transparency, environmental effectiveness and broad political support.

## Background

Global forests can play a pivotal role in preventing dangerous climate change. Net forest cover increment in most temperate forests currently leads to carbon sequestration [1]. Also, the recent increase in CO_2 _concentrations and warming stimulates carbon uptake in temperate and boreal forests, although this will not continue indefinitely [2]. Deforestation, however, still occurs at a large scale in tropical and sub-tropical regions. Global carbon emissions from tropical deforestation and land-use change ranged from 0.8 to 2.4 Gt C yr^-1 ^for the 1990s [3-5], accounting for 12–28% of the total annual anthropogenic greenhouse gas emissions [6]. The thirteenth conference of the parties (COP 13) of the United Nations Framework Convention on Climate Change (UNFCCC) in December 2007 at Bali provided a mandate to include measures for emission reductions from deforestation and forest degradation (REDD) in the climate change mitigation framework from 2012 on [7]. Recent scientific and policy analyses paint an optimistic picture on the feasibility of such a REDD scheme [8-11].

One of the main remaining challenges on the way to an effective REDD mechanism is the choice of the methodology to set the so-called baseline or reference scenario. All climate protection activities through emission reduction schemes under the Kyoto Protocol must prove that they have a positive net effect on the global carbon cycle [12]. This requires the establishment of an appropriate baseline scenario, which describes the future emission pathway without any climate protection measures. Hence, such baselines are crucial to measure the emission reduction performance and consequently to negotiate meaningful deforestation emission reduction targets. Up until now, both developing countries (the so-called Non-Annex-1 countries under the Kyoto Protocol) and industrialized countries (Annex-1 countries) lack national emission baselines against which additional reductions can be established and rewarded [13]. Therefore, the establishment of feasible, transparent and sound deforestation emission baselines and accounting rules remains one of the key tasks to effectively implement the REDD regime. While several baseline approaches have been proposed independently, none of them has gained broad political and scientific acceptance. A comprehensive comparison of the advantages and disadvantages of the existing baseline approaches has so far not been carried out for the national level. Thus, an assessment of REDD baseline approaches towards their applicability for a future UNFCCC policy context is urgently needed.

This paper evaluates four different baseline methods by comparing their environmental, political, economic and technical applicability. We compare three methods based on linear extrapolation of historical deforestation emission trends and one method based on a dynamic land-use model.

We first introduce and justify the chosen baseline methods. In a second step we explain our methodological approach of combining case study information and expert surveying in a weighted multi-criteria analysis. Subsequently, we assess the methodological limitations, discuss the results with regard to strengths and weaknesses of the different approaches and compare them to recent REDD policy developments. Finally, we show that the success of future REDD mechanisms will be strongly shaped by the selected baseline method and provide some policy recommendations.

### Overview on current deforestation baseline approaches

We distinguish two different categories to establish a deforestation baseline: retrospective and prospective approaches. Retrospective baseline methods assume a linear trend by extrapolating deforestation emissions rates from a historical reference period into future commitment periods. Due to the high annual variability of carbon emissions from tropical deforestation [14], most scientific analyses recommend using averages over longer past reference periods instead of single reference years. Prospective baseline methods anticipate the future behaviour of land-use change, often by understanding the drivers of past trends, to predict deforestation rates and locations [15]. Such models are similarly based on historical deforestation data, but specifically project future developments of e.g. demographic, economic and technological variables leading to specific infrastructure, energy and food demands that drive land use change [16].

### Retrospective methods: historical extrapolation

Historical baselines are calculated by extrapolating the mean relative rate of deforestation and its associated emissions over a past reference period linearly into the commitment period. For this study we applied three retrospective approaches. The chosen baseline methods represent the main methodological approaches discussed under the UNFCCC REDD process. We labelled them Simple Historical Approach (SiHA), Spatial Historical Approach (SpHA) and Joint Research Centre Approach (JRCA).

In SiHA historical relative deforestation rates are based on existing gross forest cover data from the FAO Forest Resource Assessment [17] for three points in time: 1990, 2000 and 2005. Spatial data is not available to further quantify these rates. For the conversion calculation from deforestation rates into carbon emissions, average global biomass and carbon default factors are used [18].

In SpHA historical relative deforestation rates are based on globally consistent satellite imagery of forest cover for the period 1990 to 2005. These images are taken from at least three points in time to determine forest area changes [19]. For the conversion calculation from deforestation rates into carbon emissions, regional or local data on forest area change, biomass and carbon stocks can be incorporated.

The JRCA approach is proposed by Mollicone et al. [20], who also suggest calculating the baseline from satellite imagery from the period 1990 to 2005, but furthermore introduce a method to distinguish intact forest, non-intact forest and non-forest land [21]. The related area changes of subsequent satellite images within this period determine the relative rate of deforestation and forest degradation. The approach establishes a national and global relative baseline rate of forest conversion, which is compared to the actual land-use change in the commitment period. If the national baseline rate is less than half of the global baseline rate in the reference period, then the difference is accounted as avoided forest conversion in the commitment period. If the national baseline rate is higher than this global threshold, the emission reductions occurring below the national baseline in the commitment period are accounted for (similar to the SiHA and SpHA approaches). Deforestation and carbon values are separated according to forest type, forest sub-category and forest conversion type.

### Prospective methods: dynamic spatial land-use modelling

Since the 1990's modeling of land-use and land-cover change (LUCC) has developed rapidly [22]. Land-use change and deforestation can be modeled using analytical, regression and simulation models [23]. Simulation models assess the interactions between drivers and are often spatially explicit. Depending on their purpose LUCC simulation models use a sweep of different methods including suitability mapping, genetic algorithms, neural networks, scenario analysis, expert opinion, public participation or agent-based modelling [24]. The ability of these LUCC models to combine spatial and non-spatial forest cover and driver data make them especially suited to be assessed as REDD methodology in this analysis.

A number of these models have been compared in several studies. Gonzalez [25], for example, assessed the behavior of the Geographical Modeling (GEOMOD) and Forest Restoration Carbon Analysis (FRCA) models in providing deforestation baselines, however only for the local scale. Pfaff [26] briefly compared deforestation baseline methods for project as well as national level. In the last category, he investigates the suitability of simulation models such as Computable General Equilibrium (CGE) models and more process-based spatial models, such as GEOMOD and CLUE (Conversion of Land Use and its Effects model) [27]. He concludes that CGE models are not appropriate, since their data and modeling requirements are inappropriately demanding for the national level. The advantage of GEOMOD and CLUE is their ability to model land use change spatially. Their disadvantage, however, is the requirement to exogenously define the deforested area [26]. Brown et al. [15] compared the Forest Area Change (FAC) model, the Land Use and Carbon Sequestration (LUCS) model and GEOMOD for simulating deforestation trends at the regional scale. Only GEOMOD provided results that could be used for dynamic deforestation determination under different driving factors, but GEOMOD only predicts the location of land-use change and not the quantity. The dynamic model on conversion of land use and its effects (CLUE-S) was chosen as an exemplary dynamic baseline approach for this study. It is a similar to GEOMOD but it yielded more promising results in its applications [24] and provided more comparable data to other model applications for the purpose of this study. Additionally, the model has been applied to more than 50 countries in Europe, Asia, and Latin America.

The CLUE-S model [28] explores quantitative, spatially explicit, multi-scale pathways of land-use changes through the determination and quantification of the most important driving forces. These factors are divided into bio-geophysical and human drivers of land use, based on information of land use images and socio-economic data. CLUE-S can be used both to track past land-use changes and to simulate them under different development scenarios into the near future [27]. This results in maps providing the location and quantity of future land-use changes including deforestation. If carbon values are assigned to the changes in different forest classes, the resulting change of the model can be translated into locally differentiated baseline emissions. The reductions compared to the projected deforestation emission rate in the commitment period can then be gratified. For this analysis CLUE-S case study data for the period between 1990 and 2005 was targeted to allow the comparability with the historical baseline methods.

## Methods

**Figure 1:**
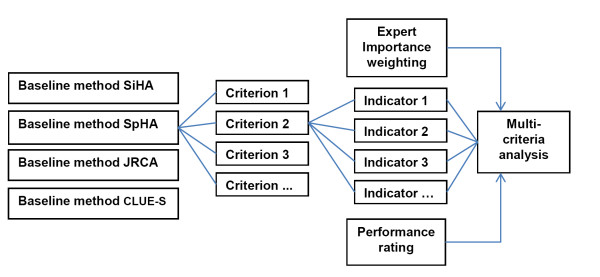
**Schematic overview**: The figure illustrates the different steps in our analysis. First, the baseline methods were classified into four approaches. Each approach was evaluated by the same set of criteria and indicators for their political, ecological, economic and technological performance. For each indicator every baseline method received a performance rating score, which was subsequently adjusted by an Expert Importance Weighting score in a multi-criteria analysis.

Figure 1 illustrates the different steps in our analysis. First, the baseline methods were classified into four approaches (i.e. SIHA, SpHA, JRCA and CLUE-S) based on current REDD policy developments [29-31]. The goal of the study was to compare the applicability of these baseline approaches. Applicability is defined here as the ability of a baseline method to be successfully implemented under a Post-2012 REDD regime as specified by several criteria. To allow for an integrative approach, these criteria characterize the political, ecological, economic and technological performance of baseline approaches, and include environmental effectiveness, equity, transparency, cost effectiveness, economic attractiveness, data and method compatibility with existing standards and output accuracy. Each performance criterion was represented by one or more indicators.

Various sources of disciplinary literature from the field of environmental policy [32,33], land-use modelling, [15,24,34,35] and national submissions to the UNFCCC [7,30,36] were used to select criteria, indicators and methods for a baseline method evaluation. To balance, limit, and streamline the criteria selection, we identified, tagged and clustered the most common baseline method characteristics from the literature [37]. Furthermore, the choice of indicators was evaluated iteratively to match the available data from the existing regional case studies.

### Indicator performance scoring

While each criterion describes a single characteristic of the baseline methods, all indicators together allow to quantify the performance of these criteria (either directly or indirectly through the use of proxy indicators, c.f. Table 1). To determine political, economic and ecological performance as well as technical data requirements and availability for each baseline approach, indicators with both descriptive or performance-based properties [38] were compared.

**Table 1:** Weighted multi-criteria analysis of the performance and importance ratings

		**Importance Survey Rating**	**Performance Rating**			**Weighted MCA**				
**Nr.**	**Indicators**	Average	SiHA	SpHA	JRCA	CLUE-S	SiHA	SpHA	JRCA	CLUE-S

1	Prediction of business-as-usual emissions from deforestation	7.1	2	2	3	4	14.2	14.2	21.3	28.3

2	Estimation of leakage and permanence	7.0	1	2	2	4	7.0	14.0	14.0	28.0

3	Precision in calculating emissions from deforestation and degradation	7.2	1	2	3	4	7.2	14.4	21.6	28.7

4	Encouragement of early action	7.8	3	3	3	3	23.3	23.3	23.3	23.3

5	Co-benefit: contribution to the management of ecosystem services	7.2	1	2	2	4	7.2	14.3	14.3	28.7

6	National sovereignty over data	6.4	4	3	3	2	25.4	19.0	19.0	12.7

7	Provision of financial incentives for countries with low deforestation rates	7.5	1	1	3	3	7.5	7.5	22.6	22.6

8	Applicability in all NA-I countries	7.5	2	2	2	2	15.1	15.1	15.1	15.1

9	Clarity to policy makers	8.3	5	4	4	2	41.3	33.0	33.0	16.5

10	Dynamic baseline updating	8.4	2	2	3	4	16.8	16.8	25.1	33.5

11	Low dependence on subjective expert input	6.7	2	4	4	2	13.5	27.0	27.0	13.5

12	Low baseline data and capacity requirements and costs	6.9	5	4	3	2	34.4	27.5	20.6	13.8

13	Financial carbon benefits for host country	7.1	2	2	3	2	14.3	14.3	21.4	14.3

14	Calculation the opportunity costs of forest protection	7.4	1	3	3	4	7.4	22.0	22.0	29.4

15	Compatibility with FAO data sets and UNFCCC forest definitions	7.5	5	2	2	2	37.4	15.0	15.0	15.0

16	Compatibility with existing IPCC Good Practice Guidelines	8.5	3	4	4	4	25.5	34.0	34.0	34.0

17	High validation accuracy	8.0	1	2	2	3	8.0	15.9	15.9	23.9

	**Sum**						**297.3**	**334.7**	**372.7**	**381.1**

Overview on the weighted multi-criteria analysis scores for each baseline approach (main right column). The scores were derived by multiplying the indicator performance rating (main middle column) for each baseline method by the mean importance rating (left column). The following criteria are represented by indicators: environmental effectiveness (Nr. 1–5), international equity (Nr. 6–8), transparency (Nr. 9–11), cost effectiveness (Nr. 12), economic attractiveness of the baseline (Nr.13–14), data and method compatibility with existing standards (Nr. 15–16) and output accuracy (Nr. 17).

The objective of evaluating the applicability of the mentioned baseline methods for a future REDD regime should ideally have been achieved by applying all different baseline approaches in sample countries under various conditions. Besides the lacking resources to conduct such extensive research, it is also extremely difficult to select a representative number of countries such that none of the approaches would be favoured over the others. We therefore opted to only use readily available published case studies and data bases in combination with general literature reviews or hypothetical case studies. We consider such an approach sufficient to assess the applicability of the methods in the general policy context of REDD. Hypothetical studies were only used to account for data shortage or to reduce the apparent topical complexity. Indicators like economic revenue, for example, were based on calculations of deforestation data from the FAO [17] in combination with hypothetical carbon market prices. The different methodologies and data sources used to evaluate every indicator performance for each baseline method are summarized in Table S1 in additional file 1. To enable comparison of the heterogeneous results, we applied a robust scoring system as developed by den Elzen et al. [39]. It distinguished the indicator performance into the following scoring values: fully satisfied (5); generally satisfied (4); partly satisfied (3); poorly satisfied (2); and not satisfied at all (1).

### Indicator importance rating

To establish the political and scientific relevance of the performance analysis, an expert survey was conducted. Participants, who were approached via e-mail and telephone, were asked to rate the importance of all 17 baseline indicators used for the performance analysis for a future REDD scheme. The useable importance scores ranged from 'no importance' (1) to 'highest importance' (10), allowing the use of similar scores during the survey. In this survey 24 international scientific experts and policy makers participated out of 74 requests, resulting in a response rate of 32 percent. The approached experts represented their countries or organisations on REDD during the UNFCCC workshops of the Subsidiary Body for Scientific and Technological Advice (SBSTA). Sixteen participants were policy makers from South America, Africa, Asia and Europe. Eight of the participants were scientific experts – both from Annex-1 and Non-Annex-1 countries. To avoid biases survey participants were ensured confidentiality and questions were asked independent of the chosen reference scenario method.

### Weighted multi-criteria analysis

The indicator performance scores for each baseline method were compared in a multi-criteria analysis (MCA). Subsequently a weighted sum model [40] was applied in which the mean survey importance ratings were multiplied with the indicator performance scoring. All products were summed for each baseline method. The applicability of the baseline methods was ranked according to these factor sums in the weighted MCA. The performance and importance scores were established at the indicator level instead of the criteria level, since this allowed for the most comprehensive and detailed assessment on advantages and disadvantages of the respective methods. Based on the results, the dangers and bottlenecks as well as the comparative advantages in the currently proposed baseline methods were determined. Finally, the overall acceptance of each baseline method in a future climate regime was qualitatively assessed by comparing the results of the weighted MCA to the current submissions of the political groups to the UNFCCC on REDD baseline methodologies.

## Results and discussion

The analysis of baseline method applicability based on indicator performance and importance rating is shown in Table 1. We first compare the results of the importance rating survey and interpret the findings of the weighted MCA by discussing the advantages and disadvantages of the baseline approaches. We then justify the chosen methodological approach by assessing its strengths and weaknesses. Finally, we compare our findings to current political REDD proposals to the UNFCCC.

### Indicator importance

**Figure 2:**
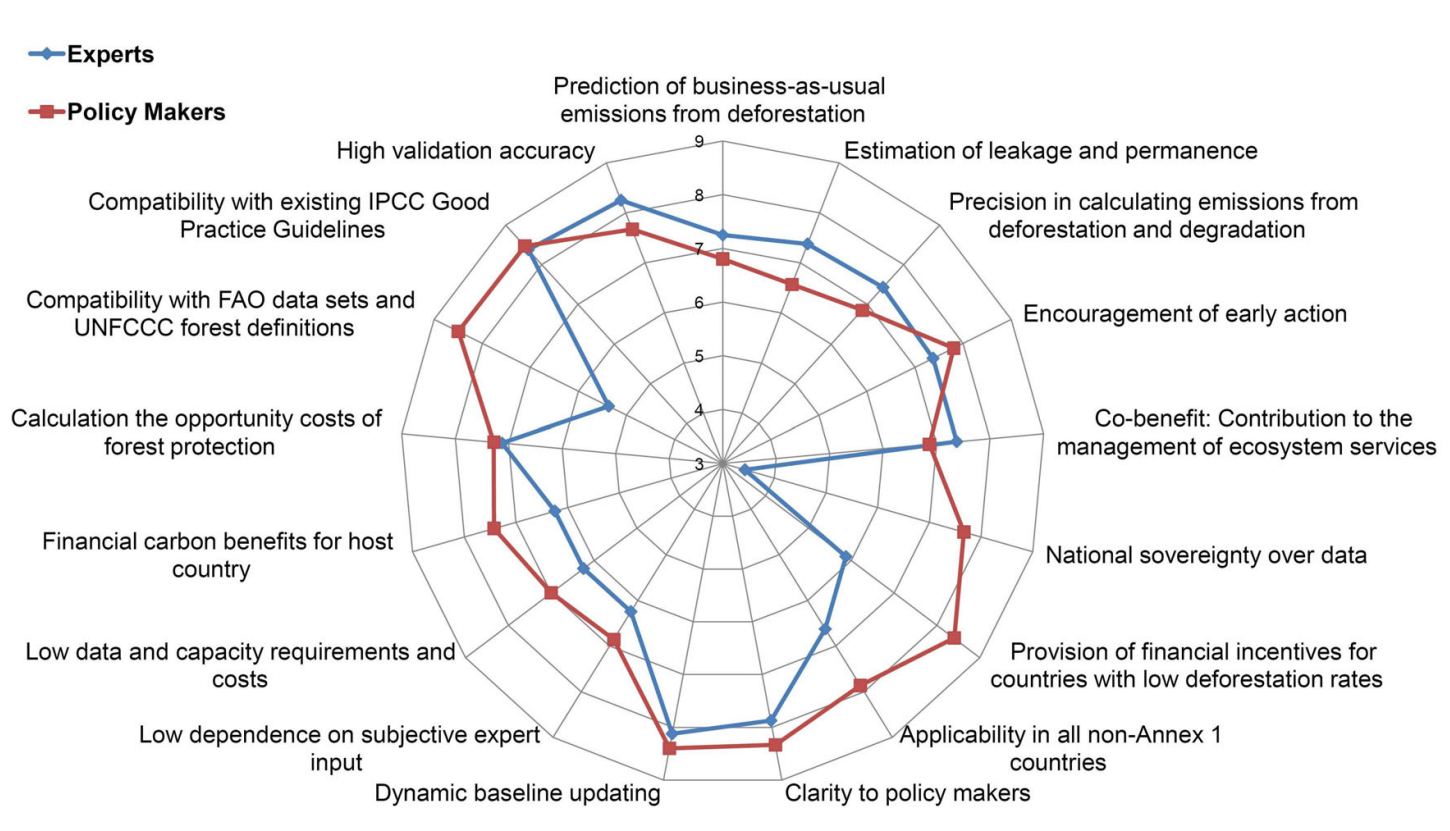
**Indicator importance rating – divided by policy makers and experts**: Mean perceived importance ratings of the indicators for a national REDD baseline method – Divided by policy makers (red colour, squared symbol) and scientific experts (blue colour, diamond symbol). Rating scores range from 1 (lowest importance) to 10 (highest importance).

**Figure 3:**
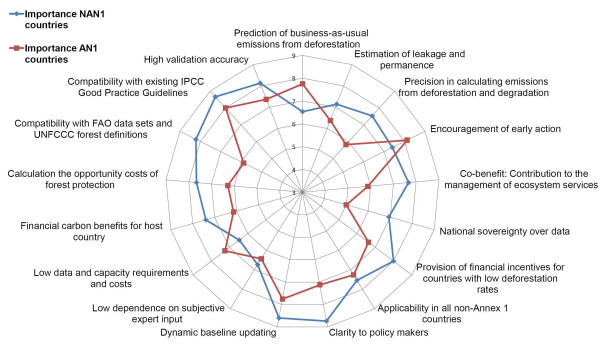
**Indicator importance rating – divided by country groups**: Mean perceived importance ratings of the indicators for a national REDD baseline method – Divided by Annex-1 (AN-1) country representatives (red colour, squared symbol) and Non-Annex-1 (NAN-1) country representatives (blue colour, diamond symbol). Rating scores range from 1 (lowest importance) to 10 (highest importance).

The survey with experts and policy makers from Annex-1 (A-1) and Non-Annex-1 (NA-1) countries revealed that the analyzed indicators are not all perceived equally relevant for a future REDD mechanism. The results of the survey are presented in Figures 2 and 3. On average all participants regarded the compatibility with existing IPCC Good Practice Guidelines, clarity to policy makersand the need for dynamic baseline updating as most important. Opposite, the national sovereignty over data, low dependence on subjective expert inputand low data and capacity requirements were rather perceived less important. As shown in Table 1, none of the listed indicators obtained an average value below 6, showing that none of them is perceived unimportant.

By splitting the survey participants into scientific experts and policy makers (see Figure 2), interesting differences become apparent. While most of the environmental indicators have higher importance for experts, elements like 'data sovereignty' and 'provision of financial incentives for countries with low deforestation rates' as well as 'compatibility with FAO data' are rated much higher by policy makers. In contrast, most experts fear the lack of transparency, if the data sovereignty is too high and doubt the scientific accuracy when relying on current FAO data.

When dividing the importance perception into A-I and NA-I country survey participants including all surveyed experts and policy makers (Figure 3), it becomes clear that elements such as 'clarity to policy makers', 'national sovereignty over data', 'provision of financial incentives for countries with low past deforestation rate' and also the 'Co-benefits: Contribution to the management of ecosystem services' have much higher importance for NA-1 countries. These results display the strong interest of NA-I countries to use a simple, equitable approach, which simultaneously addresses the biodiversity values of forests. Furthermore, the differences among A1 and NA1 countries might be influenced by the economic importance of the land use sector for developing countries. The 'compatibility with FAO data' is seen essential by NA-I representatives, since most of them are currently not able to provide alternative databases. The only element, which is viewed considerably more important by A-I countries is the 'prediction of baseline emissions from deforestation'. This is related to the concern of industrialized countries that NA-I countries might produce 'hot air' credits and thus distort the carbon market.

### Comparing the baseline approaches

**Figure 4:**
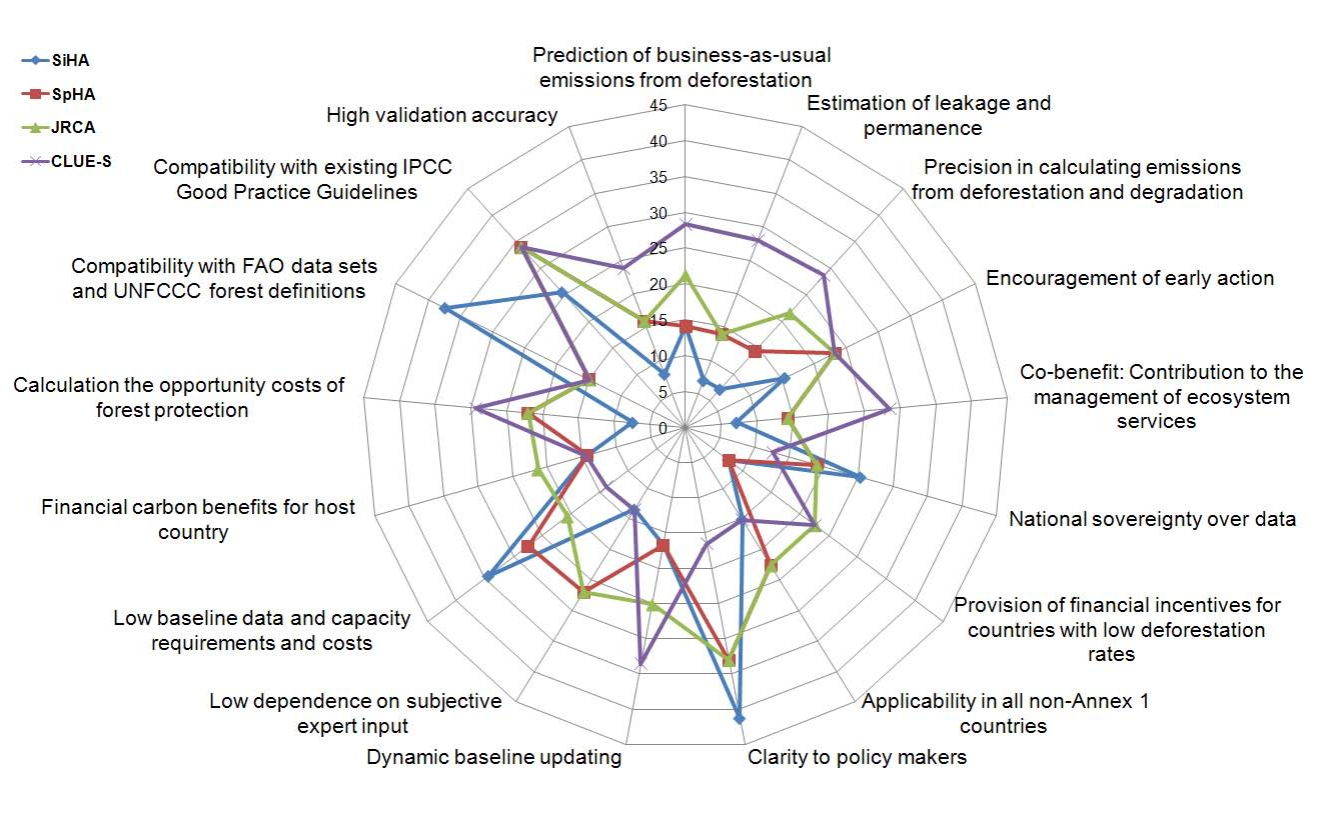
**Weighted multi-criteria analysis**: Graphical representation of the weighted multi-criteria analysis scores for each baseline approach. The further the points are from the centre, the more suitable the method is. The scores were derived by multiplying the indicator performance rating (1 = lowest performance to 5 = highest performance) for each baseline method by the mean importance rating for each indicator – Divided by Simple Historic Approach (SiHA, blue colour, diamond symbol), Spatial Historic Approach (SpHA, red colour, squared symbol), Joint Research Centre Approach (JRCA, green colour, triangle symbol) and CLUE-S Modelling Approach (CLUE-S, purple colour, cross symbol).

The importance ratings from the scientific experts and policy makers were multiplied with the performance scores into a weighted MCA scoring (see Table 1). The results show that the CLUE-S approach and JRCA have the highest weighted sum rating. However no single baseline method is clearly superior for every indicator. Rather, each baseline method has its specific strengths and weaknesses, which are graphically displayed in the spider-net diagram in Figure 4.

We now discuss the main performance results of each baseline approach in relation to their rated importance and display the related weighted MCA scorings in parenthesis. SiHA shows the best performance for indicators such as compatibility with existing FAO data (37.4) and clarity to policy makers (41.3). Related to this, the strengths of SiHA are its low data and capacity requirements (43.4) and maintained national sovereignty over data (25.4). SiHA is thus the most technically feasible option. However, the weak performance of political and environmental indicators considered important makes this baseline approach rather not recommendable. SiHA could not help in calculating the opportunity costs of REDD (7.3) and would not provide incentives for countries with low past deforestation rates (7.5), making it economically and politically unattractive. Our case study calculations showed that linear historical deforestation rate extrapolations for the business-as-usual scenario can yield huge errors (14.2).

SpHA is characterized by its moderate performance ranking, providing a simple and technically feasible baseline approach. It is not necessarily reliant on heterogeneous data sources or subjective expert input (27.0), but could use a homogenous satellite image interpretation method. It has nevertheless a few striking shortcomings, which are often similar to SiHA. Especially the lack to provide financial incentives for countries with low deforestation rates (7.5) would disregard their right of development and thus appear politically and economically unattractive to the concerned Non-Annex-1 countries. SpHA also lacks the ability to predict future non-linear business-as-usual emissions (14.2) and provides no solution to incorporate degradation emissions (14.4). Consequently, its environmental effectiveness seems questionable.

JRCA indicates medium to high applicability for most indicators. It yields good performance for all importantly ranked indicators, except the compatibility with FAO data (15.0), which also applies to SpHA and CLUE-S. The strengths of JRCA are clearly its financial outcomes. It offers the highest economic profitability of all baseline methods because the baseline calculation method of expected deforestation provides financial incentives for countries with low deforestation rates. This has high relevance to Non-Annex-1 policy makers (21.4). It reveals also a disadvantage of this method. A default minimum deforestation rate assumption of half the global average could overestimate the actual emissions for many countries, leading to inflationary issuing of credits, which would lower carbon credit prices and ultimately threaten the ecological effectiveness of the regime.

Consequently, all historic approaches have a methodological disadvantage in common: Besides our case study calculations other research stresses the non-linear rates change of deforestation for many countries, often moving towards forest transitions [13,41]. In the long run a lower relative weight of the primary sector in developing economies can be expected, leading to decreased pressure on land, and therefore less forests to be converted [42]. Consequently, linear extrapolations potentially overstate the business-as-usual emissions, while delivering no incentive for countries with low deforestation histories to join a REDD regime. The political proposals aimed at using individually set emission reduction targets instead of baselines cannot solve this dilemma. A scientific basis for the negotiation of a quantified target requires predicting the deforestation potential, otherwise non-transparent political bargaining might determine inappropriate targets.

A prospective approach, such as the CLUE-S model, demonstrates high applicability as baseline method in providing environmental effectiveness. By calculating the quantity, location and timing of deforestation and related GHG emissions, it serves best for determining leakage and permanence (28.0), even if this plays only a role for sub-national monitoring. This feature might also be utilized for managing ecosystem services such as biodiversity protection (28.7). Dynamic models have also economic advantages by enabling financial incentives to countries with low deforestation rates and high deforestation potential as well as allowing calculating the opportunity costs of REDD in a spatially differentiated manner (29.4). However, the low national sovereignty over data is also a typical feature of a complex model approach (12.7). It is closely related to the high data, modelling and capacity requirements (13.8) involving subjective expert judgement (13.5). The related low clarity to policy makers and necessary assumptions on driver behaviour reduce its transparency (16.5). Interestingly, these disadvantages do not change the ranking order for the weighted MCA, even if only Non-Annex 1 representatives are considered. This might be explained by the superior performance for other indicators. Also the counterintuitive moderate indicator rating for 'Low baseline data and capacity requirements and costs' by NA-1 country representatives contributes to this result.

The advantage of using model-derived baselines compared to extrapolated historical deforestation baselines is potentially founded in their ability to display non-linear factors of deforestation. However, the related provision of socio-economic and policy trajectories required for land-use models would allow actors to bias such data for their own advantage, for example, by ignoring a planned establishment of a national park or by proposing the establishment of a dense road network. This can lead to an overestimation of baseline deforestation emissions and thus violate the environmental and economic effectiveness of a REDD regime. Consequently, the application of dynamic modelling baselines would need to be accompanied by transparent documentation of their underlying assumptions.

The described four baseline approaches are not mutually exclusive. The historical baseline methods can provide a reference value, which might be adjusted using dynamic modelling to determine the future, country-specific reference target. In return, land-use models can utilize historical deforestation rates as quantity input. Consequently, the proposed four methods might exchange techniques and characteristics among each other during the ongoing policy process. We furthermore expect that our research is useful in defining the differences of the outlined baseline approaches, since the performance and importance evaluation can be assessed flexible according to the division into indicators.

### Methodological considerations

The combination of different methods and results using approximated scoring values for a MCA is strength and weakness at the same time. The importance ranks as well as the performance scores are simplified and might thus not be able to adequately represent quantitative and qualitative details. Moreover, the performance scoring conducted by the authors potentially suffers from subjectivity, unless the rationale of scoring is made transparent. However, the strength of the MCA to combine quantitative and qualitative results allows a comprehensive decision support for complex problem assessment. The comparison of REDD baseline methodologies presents such a case for which robust and comprehensive political guidance is urgently needed. The risk of imprecise and changeable results in multi-criteria decision making can be limited by applying sensitivity analyses on the input data [43].

We used a sensitivity analysis to test the influence of importance ratings on the final scores derived from the weighted MCA. Here, different weight distributions were calculated using averaged importance scoring or weight calculation scorings separated by expert/policy maker group as well as Annex-1/Non-Annex-1 member. The results are summarized in Figure S1 and Table S2 [see additional file 1]. For all options in the sensitivity analysis the ranks of the different baseline methods based on the summed weighted MCA scoring remained similar compared to the initial results. Thus, the importance rating can be regarded robust.

Table S3 in additional file 1 gives an overview on the deviation among summed performance scores for the different baseline methods which would lead to changing rankings. While summed ranking changes of SiHA and SpHA towards other methods require at least 5 scoring points, the difference among JRCA and CLUE-S ranking is very small. The latter indicates a rather high sensitivity of the ranking order, which has to be considered when interpreting the results.

The importance rating is closely tied to the political preferences of the survey participants and such preferences are usually subject to change. To limit the influence of pre-defined baseline method preferences, survey participants only rated the indicators without knowing which baseline methods were used in the comparison. We are also aware that there might be institutional and technical capacity differences among Non-Annex-1 countries, influencing the importance rating within this sub-group. But the heterogeneity within the subgroup ratings remained relatively low. Furthermore, the outcome of the weighted MCA can only resemble a temporary analysis since both performance and importance are subject to dynamic perceptions. Nevertheless, the selected methodology provides an anchor point for such dynamics in time to assess the strengths and weaknesses of the chosen approaches.

### Policy relevance of baseline approaches

The results of the weighted MCA were assessed using the REDD baseline proposals from various political groups. The policy analysis of UNFCCC submissions revealed the current disagreement on the choice of the proposed baseline mechanisms. This is related to the different national circumstances, data availability and national capacity, political preferences and expected revenues. However, both the political proposals and our survey results evinced the high interest of tropical countries in participating in a future REDD regime, despite their heterogeneous circumstances and preferences. Initially many country delegations proposed the application of extrapolated historical deforestation rates based on remote sensing images comparable with the SpHA [29,30,44]. In the last two to three years especially countries with low past deforestation rates such as the Congo Basin states have increasingly requested modifications to account for specific country circumstances in the baseline setting [45]. The Coalition for Rainforest Nations [46] suggests using a historic baseline with a development adjustment factor to approach this need. On the 30th meeting of the SBSTA in June 2009 participants recognized that baseline establishment could take into account historic data, national circumstances, drivers of deforestation and if appropriate adjust for expected future emission trends [47]. Dynamic land use models such as CLUE-S can better account for these requirements than any historical method.

In the Bali Roadmap agreed at COP 13 [44] the need to include forest degradation was emphasised. Thus, techniques such as proposed for the JRC approach to measure degradation provide potential solutions for a future REDD regime. Most interestingly, those baseline methods least favoured by policy makers – namely the JRCA and CLUE-S – rank highest in the overall applicability analysis for the baseline methods. Their mentioned shortcomings of low transparency to policy makers and high data requirements can explain the lacking political popularity. On a project level, however, land use models are frequently used in REDD baseline methodologies in the current voluntary carbon market schemes [15,48].

## Conclusions and recommendations

To effectively involve developing countries in the Post-2012 process, it will be essential to build a policy mechanism to reduce emissions from deforestation and degradation. The success of such a REDD mechanism will be strongly shaped by the selected baseline method. The goal of this study was to evaluate the applicability of four different REDD baseline approaches for NA-I countries in a Post-2012 climate regime by using a set of environmental, political, economic and technical criteria and indicators. The results of a comprehensive MCA were compared in light of current REDD negotiations under the UNFCCC.

The dynamic land use model CLUE-S and the JRCA yielded the highest ranks in the weighted MCA. However, this does not immediately mean that these two approaches are superior, since all have strengths and weaknesses. The performance of each method also differs for the various national circumstances. The SpHA and JRCA show a relatively balanced performance. In contrast to historical methods, spatially-explicit models like CLUE-S allow for a better estimation of non-linear deforestation trends, leakage, permanence and opportunity costs of deforestation. Their ability to incorporate the country-specific circumstances such as drivers of deforestation makes them very attractive from a financial benefit and environmental performance perspective. The complexity of nearly all LUCC models nevertheless limits their transparency and clarity to policy makers. This, in turn, might make them currently unacceptable for many developing countries as a key method for post-2012 policies.

Since most NA-1 countries will not have the necessary technical capacity and data to use advanced deforestation baseline techniques from the start of a post-2012 REDD scheme, countries should be allowed to choose the deforestation baseline methodology according to their respective situation. This could be done through the adoption of the IPCC Good Practice Guideline three-tier-approach. Countries with advanced data and research capacity can use LUCC models immediately and thus produce tier-three baselines. Data-poor countries could start by using historical extrapolation methods, such as SpHA, representing a tier-one approach. JRCA could serve as tier-two method, if the global reference level is substituted by individual national or regional deforestation potential assessments.

Baseline development for forest degradation remains a challenge for all methods. The simplified degradation detection approach under the JRCA is a reasonable alternative until proper remote sensing techniques and ground inventory data become available.

Our analysis highlights the strength and weaknesses of different baselines approaches in their applicability for a future REDD regime. It shows that a multi-tier approach will allow countries to select the baseline method best suiting their specific capabilities and data availability while simultaneously enabling broad political support.

## Competing interests

The authors declare that they have no competing interests.

## Authors' contributions

MH developed the main analysis framework and carried out the multi-criteria analysis. RL contributed on the methodology section and delivered critical inputs on the discussion and policy sections. KK contributed on the model description and in conducting the indicator performance analysis. JE contributed on the development of the indicator importance survey and the policy section. All authors read and approved the final manuscript.

## Supplementary Material

Additional file 1 **Data sources and sensitivity analysis of the MCA**. The tables and figure illustrate the data sources used to conduct the MCA illustrates the results of its sensitivity analysis. Click here for file
