# Supplementary material for: A comparison of baseline methodologies for 'Reducing Emissions from Deforestation and Degradation'
Source: Carbon Balance Manag. 2009 Jul 13;4:4. doi: 10.1186/1750-0680-4-4 (PMC2717061; doi:10.1186/1750-0680-4-4)
Supplement: Additional file 1 — Data sources and sensitivity analysis of the MCA. The tables and figure illustrate the data sources used to conduct the MCA illustrates the results of its sensitivity analysis. [file 1750-0680-4-4-S1.doc]

# Additional file 1: Data sources and sensitivity analysis of the MCA

**Table S1:** Applied methodologies and data sources used to determine the indicator performance in the multi-criteria analysis

| **Nr.** | **Indicators** | **Applied methodology** | **Data sources** |
| --- | --- | --- | --- |
| 1 | Prediction of business-as-usual emissions from deforestation | Deforestation trend extrapolation, comparison with CLUE-S case study results | [1],[2], unpublished data of [3] |
| 2 | Estimation of leakage and permanence | Literature review | [4], [5], [6], [7], [8], [9], [10], [11] |
| 3 | Precision in calculating emissions from deforestation and degradation | Hypothetical case study (emission calculation scenario), Literature review | [12], [5], [13], [1] |
| 4 | Encouragement of early action | Literature review | [14], [5], [2], [15] |
| 5 | Co-benefit: contribution to the management of ecosystem services | Literature review, Theoretical ecosystem service monitoring example | [16], [17], [18], [19] |
| 6 | National sovereignty over data | Literature review | [20], [21], [4], [22], [5], [23] |
| 7 | Provision of financial incentives for countries with low deforestation rates | Schematic pathway analysis, Forest cover change calculation | [1], [2], [5] |
| 8 | Applicability in all NA-I countries | Literature review | [24], [4], [25], [26], [27], [28] |
| 9 | Clarity to policy makers | Literature review | [4], [26], [27], [18] |
| 10 | Dynamic baseline updating | Schematic pathway analysis, Literature review | [4], [26], [29], [30] |
| 11 | Low dependence on subjective expert input | Literature review, qualitative case study analysis | [4], [26], [27], [18], [2], [1], [31] |
| 12 | Low baseline data and capacity requirements and costs | Literature review | [32], [33], [34], [4], [26], [27], [2], [12] |
| 13 | Financial carbon benefits for host country | Finance calculations based on case studies of the carbon credit benefit scenarios | [35], [8], [5], [2], [1] |
| 14 | Calculation the opportunity costs of forest protection | Hypothetical case study on opportunity costs of deforestation reduction, Literature review | [8], [2], [36], [37] |
| 15 | Compatibility with FAO data sets and UNFCCC forest definitions | Literature review | [20], [1], [12], [34], [4], [31] |
| 16 | Compatibility with existing IPCC Good Practice Guidelines | Literature review | [38], [31], [12], [39], [26], [27], [40] |
| 17 | High validation accuracy | Extrapolation of forest area prediction and observation based on case study data | [34], [31], [40], [13], [10], [41], [11], [42], [3], [18], [unpublished data of von Lüpke, 2007] |

**Table S2:** Results of the sensitivity analysis: Changes in the summed WMCA rating based on selection or rescaling of importance scoring sums. Figure 5 provides a graphical representation and further information on the chosen approach.

| **Type of importance rating modification** | Original survey rating | Averaged Rating | Selected survey ratings Policy | Selected survey ratings Experts | Selected survey ratings  A-1 | Selected survey ratings NA-1 |
| --- | --- | --- | --- | --- | --- | --- |
| **Modification label** | A | B | C | D | E | F |
| SiHA | 297.3 | 303.4 | 317.0 | 270.9 | 278.6 | 312.7 |
| SpHA | 334.7 | 325.6 | 333.2 | 302.8 | 300.5 | 333.9 |
| JRCA | 372.7 | 362.6 | 372.5 | 337.3 | 334.0 | 373.5 |
| CLUE-S | 381.1 | 377.4 | 383.9 | 360.8 | 348.0 | 389.9 |

**Table S3:** Deviation of the indicator performance sum of the weighted multi-criteria analysis among baseline methods: Each number represents the necessary scoring changes to reach indifference between the performance scoring sums of two baseline methods, which are represented by the respective row and column combinations.

| **Deviation** | **SiHA** | **SpHA** | **JRCA** | **CLUE-S** |
| --- | --- | --- | --- | --- |
| **SiHA** | 0 | -5 | -10 | -11 |
| **SpHA** | 5 | 0 | -5 | -6 |
| **JRCA** | 10 | 5 | 0 | -1 |
| **CLUE-S** | 11 | 6 | 1 | 0 |

**Figure S1 - Sum scorings of the weighted multi-criteria analysis under different importance rating modifications:**


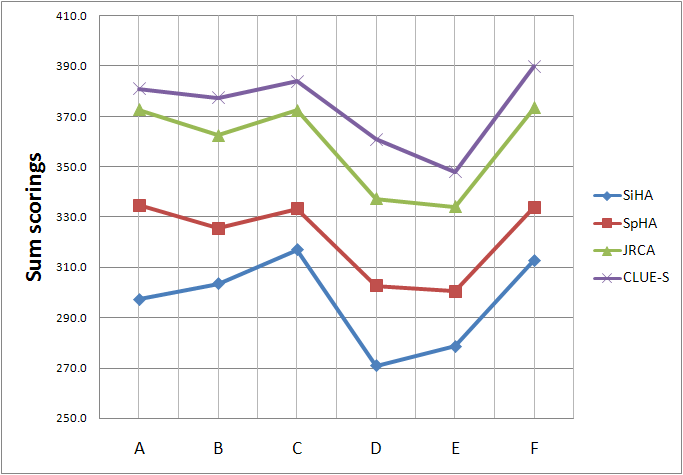


The weighted sum scorings for every baseline approach are derived by summing up the product of each indicator performance score with each indicator importance rating. The figure displays the sensitivity of the multi-criteria analysis under the following importance rating modifications: Original survey rating (A), averaged importance rating (B), selected policy maker survey rating (C), selected expert survey rating (D), selected Annex-1 country rating (E), selected Non-Annex-1 country rating (F) – Divided by Simple Historic Approach (SiHA, blue colour, diamond symbol), Spatial Historic Approach (SpHA, red colour, squared symbol), Joint Research Centre Approach (JRCA, green colour, triangle symbol) and CLUE-S Modelling Approach (CLUE-S, purple colour, cross symbol).

# References (Additional file):
